# Physician Empathy as Perceived by Parents of Children with Psychiatric Disorders: A Quantitative Analysis of Pediatric Consultations

**DOI:** 10.3390/jcm14197108

**Published:** 2025-10-09

**Authors:** Elisabeta-Oana Avram, Lavinia-Alexandra Moroianu, Cecilia Curis, Oana-Maria Isaila, Elena-Alexandra Bratu, Iulian Bounegru, Alexandru Paul Baciu, Eduard Drima

**Affiliations:** 1 Doctoral School of Biomedical Sciences, Dunărea de Jos University, 800201 Galati, Romania; titeoana@yahoo.com (E.-O.A.); alexandra.brt99@gmail.com (E.-A.B.); 2 Medical Department, Faculty of Medicine and Pharmacy, Dunărea de Jos University, 800201 Galati, Romania; 3 Department of Legal Medicine and Bioethics, Faculty of Dental Medicine, “Carol Davila University” of Medicine and Pharmacy, 020021 Bucharest, Romania; oana_maria.isaila@yahoo.com; 4 Competences Centre: Interfaces-Tribocorrosion-Electrochemical Systems, Dunărea de Jos University, 47 Domnească Street, 800008 Galati, Romania; iulian.bounegru@ugal.ro; 5 Clinical Medical Department, Faculty of Medicine and Pharmacy, Dunărea de Jos University, 800201 Galati, Romania; baciu91@yahoo.com (A.P.B.); drima_edi1963@yahoo.com (E.D.)

**Keywords:** empathy, pediatric psychiatry, parents, ASD, physician–patient communication, satisfaction, PCA

## Abstract

**Background**: Clinician empathy is associated with family satisfaction and reduced anxiety, but quantitative data from the parents’ perspective in pediatric psychiatry are limited. **Objective**: To assess parent-perceived physician empathy in pediatric psychiatry consultations and explore its associations with clinical and demographic factors. **Methods**: Cross-sectional, consecutive sample of parents attending an outpatient pediatric psychiatry clinic (*n* = 163 parents). A 10-item behavioral empathy scale (range 10–40) was used. Analyses included reliability testing, group comparisons, correlations, OLS regression, and exploratory PCA. **Results**: The mean total empathy score was 34.5 (SD 4.2); most parents rated physicians as highly empathic (65%). Parents of children with ASD reported lower empathy compared to those with anxiety/depression. Empathy increased modestly with child age and was associated with a calmer state at the end of the visit. PCA suggested exploratory evidence of potential subdimensions, including child-centered communication and listening/facilitation. **Conclusions**: Parent-perceived empathy in this sample was generally high; however, behaviors that directly involve and facilitate the child (listening, encouraging questions) may need strengthening, particularly for children with ASD. Results should be interpreted in light of the single-center design, the absence of a recorded participation rate, parent-proxy reporting, and the exploratory nature of the PCA.

## 1. Introduction

Clinical empathy is the physician’s ability to understand and communicate the patient’s perspective, with measurable effects on satisfaction, anxiety, and adherence. In pediatrics, parents’ perceptions strongly influence care experience. Beyond individual clinician skills, empathic perception is also shaped by systemic and organizational factors. Studies show that time pressure, outpatient volume, and institutional support for communication training can significantly influence how empathy is expressed and perceived in consultations [1,2]. Recent pediatric research further suggests that smoother empathic interactions are observed in clinical settings with lower workload and stronger structural continuity, such as pediatric rheumatology or general surgery, compared with high-pressure environments like psychiatry or emergency care [3,4]. This broader view underscores that empathy is not only an individual trait but also an emergent property of the healthcare context.

In pediatrics, the communication pathway is often mediated by the caregiver (parent/guardian), and their expectations and perceptions can directly influence satisfaction, understanding of recommendations, and information retention after the consultation. Studies from pediatric services show that perceived empathy by caregivers is a major determinant of satisfaction—more significant even than demographic characteristics of the family or the physician—highlighting empathy’s role as a central link in the quality of the clinical relationship in child care [5,6,7]. Prior research has shown that caregiver expectations and perceptions influence satisfaction, adherence, and cooperation in pediatric and child psychiatry [7,8,9].

A large cross-sectional study in Israel revealed that over 55% of parents are concerned about psychosocial issues. However, fewer than half actually discussed these concerns with pediatricians, and nearly 60% believe such issues fall outside the pediatrician’s role. This emphasizes how parental expectations can affect the level of empathetic engagement and conversations about mental health.

Pediatric care increasingly requires multidisciplinary approaches that integrate not only biomedical but also psychological and communicative dimensions.

Empathy is highly relevant in pediatric psychiatry, where anxiety, avoidant behaviors, communication difficulties, and the need for predictability can amplify the vulnerability of both the child and their family. Differences between children with and without autism spectrum disorders indicate that communication should be explicitly adapted to neurodevelopmental particularities to ensure a positive experience. Recent data suggest that in neurodevelopmental care, children tend to evaluate their relationship with the physician more critically than parents or clinicians do, reinforcing the need for visible empathic behaviors directed specifically at the child [10,11,12].

In interpreting ASD-related differences, we adopt the **double empathy problem** perspective: communication breakdowns can be bidirectional, arising from mismatches in communicative style, sensory processing, and expectations between autistic individuals/families and clinicians, rather than reflecting clinician shortcomings alone. This framework helps explain why parent-reported empathy may be lower in ASD when usual routines are not adapted (e.g., predictable sequencing, concrete language, additional processing time, reduced sensory load) and shifts the focus toward mutual accommodation and context-sensitive adaptations that make empathic intent visible to the child and family [13,14]. As a converging example from pediatrics, caregiver-perceived empathy in orthopedic clinics was strongly associated with feeling carefully listened to (*p* < 0.001) and respected (*p* = 0.007), underscoring the centrality of attentive listening in empathic perception [6].

Within the Transactional Model of Physician Compassion, a systematic review identified numerous factors that influence empathy and compassion, offering a conceptual framework that can inform the interpretation of predictors in our regression analyses [15].

In this context, measuring the physician’s clinical empathy through the parent’s perspective provides a practical insight into the quality of the doctor-child relationship. It highlights areas where empathetic behaviors can be improved. Basing this approach on validated frameworks (such as operational definitions and established instruments) and linking it to outcomes meaningful to the family (like satisfaction, anxiety, and the child’s condition at the end of the consultation) can lay a strong foundation for educational initiatives and organizational enhancements in pediatric psychiatry. The Transactional Model of Physician Compassion highlights how intrapersonal, interpersonal, and contextual factors shape empathic behaviors in clinical encounters [9,16].

Although clinical empathy has been widely examined in pediatrics and validated, quantitative assessments specific to pediatric psychiatry remain scarce. Much of the available work addresses broader caregiver–clinician communication or non-psychiatric contexts (e.g., surgical/orthopedic clinics) where empathy has been measured with established tools, but not within routine child and adolescent mental-health consultations. Moreover, when empathy is assessed via parent-proxy alone, scores may diverge from those reported by children or clinicians due to differences in perspective, health literacy, and social-desirability/halo effects. These gaps underscore the need for psychiatry-specific validations and multi-informant approaches that triangulate parent, child, and clinician reports [17,18,19]

While there are pediatric validations of patient-reported empathy tools (e.g., the Visual CARE Measure in pediatric emergency departments), parent-reported proxy applications in child psychiatry remain rare and fragmented; many studies focus on satisfaction or communication strategies rather than empathy operationalized and measured in a standardized way during mental health consultations [7,17].

Recent methodological reviews highlight the heterogeneity in measuring empathy (tools, perspectives, outcomes), which complicates extrapolation to CAMHS; in parallel, pediatric literature documents high-emotional-load situations (e.g., intensive care conferences) where physicians’ empathic responses are analyzed, but again outside the framework of outpatient pediatric psychiatry [7,20].

Ultimately, professional guidance clearly supports empathetic communication as a key skill in pediatric mental health practice, but it still lacks a strong base of quantitative studies focused on parents’ perceptions during current (everyday) consultations. This gap between recommendations and direct quantitative evidence motivates the present analysis.

Despite substantial evidence on empathy in pediatrics, few studies focus specifically on outpatient child psychiatry. Parental perceptions provide a unique proxy for the quality of physician–child interaction in this vulnerable population, justifying the present analysis.

Beyond individual clinician–child exchanges, perceptions of empathy are also shaped by psychosocial, cultural, and organizational contexts. Caregiver expectations, health literacy, and culturally grounded communication norms can modulate how the same behaviors are interpreted, while service-level factors (e.g., clinic workflow, time constraints, continuity of care) influence opportunities to display empathic behaviors. In neurodevelopmental care, the “double empathy problem” highlights that communication breakdowns may be bidirectional—arising from misaligned communicative styles between clinicians and autistic individuals—rather than solely from clinician shortcomings. These perspectives are particularly salient in child and adolescent mental health, where anxiety, sensory load, and the need for predictability intersect with family stress and sociocultural values. Accordingly, we interpret parent-reported empathy within this wider ecological frame and examine how observable behaviors (clear explanations, direct child address, active listening) relate to family-relevant experience indicators [13,17,21,22,23,24,25].

### 1.1. Primary Objective

1. To quantify the level of perceived empathy of the physician in pediatric psychiatric consultations, using a total composite score (theoretical range 10–40) derived from the 10 items of the empathy scale (A–D recoded 4–1).

### 1.2. Secondary Objectives

2. To identify differences between subgroups in terms of perceived empathy, by comparing the total score between:

- diagnostic categories (clinically aggregated groups),
- parent/relative gender,
- parent educational level,
- residential environment (urban vs. rural).

3. To estimate correlations between perceived empathy and:

- child age (years, continuous variable),
- child condition at the end of the consultation (ordinal scale: “understood and calm” ↔ “still scared”).

4. Identifying independent predictors of perceived empathy (total score) through regression models (e.g., OLS), having as potential predictors: diagnosis, child age, parent gender, educational level, and residential environment.
5. Exploring the dimensionality of the empathy scale (exploratory factor analysis/PCA) to assess the existence of a general factor and/or specific dimensions (e.g., explanation, active listening, direct addressing of the child), as well as estimating the internal reliability (Cronbach’s α) of the instrument.

## 2. Methodology

### 2.1. Design and Setting

We conducted a single-center, cross-sectional observational study in the Outpatient Pediatric Psychiatry Clinic of the Braila County Emergency Clinical Hospital. During all outpatient sessions between June and August 2025, research staff approached all eligible caregivers consecutively at check-out, immediately after the consultation. The treating clinician was not present during approach or survey completion, and only one response per family/episode was accepted. Questionnaires were self-administered on site and returned before departure. We did not prospectively log the full denominator of approached/eligible families, so a precise participation rate cannot be reported. When non-participation was volunteered, the most common reasons were time constraints and a preference to defer survey completion. We report the analyzed N for each table/model and use complete-case analyses where applicable. This consecutive recruitment strategy and the separation of clinical care from data collection were intended to reduce selection and social-desirability biases, but residual selection bias remains possible.

#### 2.1.1. Participants

Eligible participants were the parents/legal guardians of children aged 2–17 years who had a consultation in the service during the study period. 

**Inclusion criteria**: understanding Romanian, providing informed consent, and completing the questionnaire after the consultation. Only one response per family/clinical episode was accepted. The final analyzed dataset included *n* = 163 respondents. 

**Exclusion criteria** were: children outside the 2–17 years age range, caregivers who were not legal guardians, inability to provide informed consent, or language barriers that prevented accurate completion of the questionnaire. Families were also excluded if the consultation involved acute emergencies where the caregiver could not reasonably complete the survey.

#### 2.1.2. Instruments and Variables

##### The Questionnaire

The questionnaire had two sections:

6. Demographic/clinical data: child’s age (years), child’s sex, main diagnosis (free text later aggregated into clinical groups), residence (urban/rural), parents’ sex, and educational level.
7. Perception of the physician–child/family interaction, including the empathy scale (10 behavioral items) and an experience anchor item (the child’s condition at discharge/leave).

##### Empathy Scale: Development and Scoring

A de novo 10-item behavioral checklist was developed for parent-proxy use in outpatient child and adolescent psychiatry, focusing on observable empathic behaviors (e.g., explaining steps in child-friendly language, direct address to the child, attentive listening, facilitating questions, acknowledging emotions). The initial item pool (21 statements) was generated from pediatric communication frameworks and constructs commonly represented in validated empathy measures (e.g., CARE, JSPE/JSPPPE), then reviewed by an interdisciplinary clinical panel (pediatric psychiatry/pediatrics/communication) for face and content validity (relevance, clarity, redundancy). The final 10-item version employed a 4-point ordinal scale (A–D), with the response options recoded as 4–1 (higher = greater perceived empathy), resulting in a total score equal to the sum of the items (10–40). For the fear-response item, options were harmonized onto the same metric (from “soothes and explains” = 4 to “ignores” = 1). The measure is pragmatic and setting-specific; full psychometric validation beyond internal consistency and exploratory dimensional checks has not yet been completed.

##### Empathy Scale

The 10 items capture observable empathic behaviors (e.g., explaining the steps, speaking in a way the child can understand, active listening, addressing the child directly, managing fear, showing concern for the child as a person).

- Four-point scale (A–D) responses, recoded: A = 4, B = 3, C = 2, D = 1 (higher score = greater perceived empathy).
- For the item “How does the doctor react when the child is scared?”, the options were harmonized on the same metric: from “Soothes and explains” = 4 to “Ignores” = 1.
- Total empathy score: sum of the 10 items (range 10–40).

##### Auxiliary Variables

- Child’s state at departure: ordinal scale 1–4, recoded in ascending order (1 = “still frightened”, 4 = “understanding and calm”).
- Diagnostic groups (aggregation of open-ended responses): ASD, ADHD, Anxious/Depressive, Language/Learning; categories with very low frequencies were merged into “Others” for analytical stability.
- Demographic predictors: parent gender (F/M), educational level (no education → higher education), residence (urban/rural).
- Child’s age: numerical (years).
- Contextual variables such as consultation length, clinician caseload, and prior therapeutic relationship were not recorded and thus could not be modeled.

##### Data Collection Procedure

Immediately after the consultation, research staff invited the accompanying parent/guardian to complete a self-administered questionnaire on site. Participation was anonymous; no personally identifiable information was collected. To limit response bias, the treating clinician was not present during completion and had no access to individual responses thereafter. Only one response per family/episode was accepted.

#### 2.1.3. Ethical Considerations

The study was approved by the Clinical Studies Ethics Committee of Braila County Emergency Clinical Hospital (Favorable Opinion No. 1/9 April 2025; Registration No. 13887/9 April 2025). Informed consent was obtained from all parents/legal guardians. Data were collected anonymously and managed in accordance with current legislation, including GDPR.

#### 2.1.4. Data Management and Quality Control

Responses were exported to a tabular dataset and screened for internal consistency, duplicate entries, and valid value ranges. Items from the empathy scale had no missing data; for variables with occasional missingness, multivariable analyses were performed on complete cases. All recoding steps (including diagnosis grouping) were documented in a prespecified coding dictionary. Free-text primary diagnoses reported by parents were subsequently mapped to ICD-10 categories and collapsed into the following analytic buckets: autism spectrum disorder (ASD), attention-deficit/hyperactivity disorder (ADHD), anxiety/depression, and language/learning disorders. Low-frequency diagnoses were merged into an “Others” category to preserve analytical stability.

#### 2.1.5. Statistical Analysis

##### Descriptive Statistics

Continuous variables: mean (SD), median, min-max, quartiles; categorical variables: frequencies (%).

##### Scale Reliability

Cronbach’s α; corrected item-total correlations and α if item deleted.

##### Group Comparisons

**Binary** (parent sex; urban/rural): Welch’s t-test (unequal variances), with mean difference, 95% CI, and Cohen’s d.

**Multicategorical** (educational level; diagnostic group): one-way ANOVA; assumptions evaluated through residual inspection and Levene’s test; Bonferroni post hoc; partial η^2^ as effect size.

##### Correlations

Pearson (r) between empathy score and the child’s age.

Spearman (ρ) between empathy score and departure status (ordinal 1–4).

##### Multivariate Modeling

Linear regression using OLS was conducted with the empathy score as the outcome variable. Predictors included diagnostic group (with ADHD as the reference category), child’s age, parent sex, educational level, and residence. The report presents coefficients β, standard errors, 95% confidence intervals, *p*-values, and R^2^. Model assumptions were visually checked for linearity, homoscedasticity, and residual normality. Sensitivity analyses involved calculating robust standard errors (HC3) when heteroscedasticity was detected. VIF was used to assess multicollinearity.

##### Factor Analysis (PCA)

To explore the dimensionality of the 10-item empathy scale, we employed principal component analysis (PCA) rather than exploratory factor analysis (EFA). PCA was selected as a pragmatic approach because the study aimed to obtain an initial indication of the scale’s overall structure and variance distribution, rather than to model latent constructs with unique error terms. This choice also reflects the relatively modest sample size and the borderline factorability of the item correlation matrix, for which PCA offers more stable estimates than EFA. Findings are interpreted as exploratory evidence only, and future studies with larger samples should apply EFA and confirmatory factor analysis (CFA) to test the latent structure more rigorously.

##### Reporting

Two-tailed tests, α = 0.05; 95% CI; number of observations used in each analysis is reported.

#### 2.1.6. Software

Analysis was performed in R 4.5.0 (packages: tidyverse, psych, car, effectsize, parameters, performance, factoextra), GraphPad Prism 9.3.0 (descriptions, t-test/ANOVA, correlations, graphs), and JMP Pro 17 (PCA, regression, plots). Settings (α, post hoc corrections, correlation type, and missingness treatment) were kept consistent across platforms for reproducibility.

## 3. Results

### 3.1. Sample Characteristics

The analyzed sample comprised 163 parent–child dyads. Children’s mean age was 10.35 ± 4.04 years. Residence: Urban 93/163 (57.1%); Rural 70/163 (42.9%). Respondent (caregiver) gender: Women 149/163 (91.4%), Men 14/163 (8.6%). Education: No schooling 11/163 (6.7%), Primary 16/163 (9.8%), Lower secondary 35/163 (21.5%), Upper secondary 74/163 (45.4%), Tertiary 27/163 (16.6%). Diagnostic groups: Language/Learning 48/163 (29.4%), ASD 38/163 (23.3%), ADHD 34/163 (20.9%), Anxiety/Depression 24/163 (14.7%), Others 13/163 (8.0%), IDD/DD 4/163 (2.5%), Conduct/Behavior 2/163 (1.2%). The sample characteristics are presented in Table 1, Table 2 and Table 3.

### 3.2. Performance of the Empathy Scale

**Figure 1:**
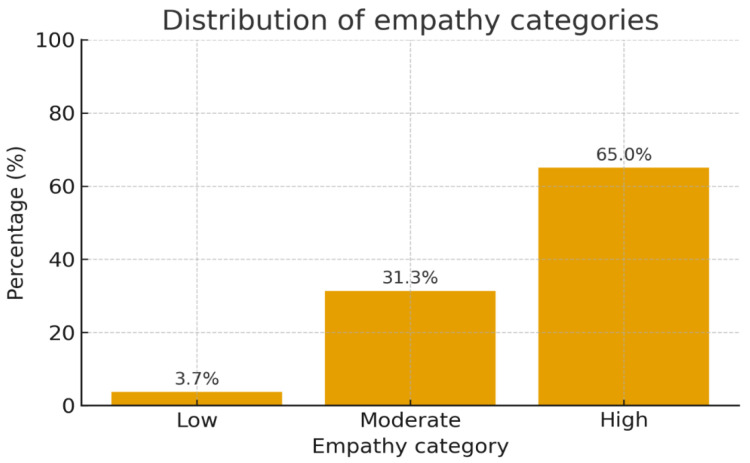
Distribution by empathy categories.

The overall perceived empathy score was high, averaging 34.51 (SD 4.16) with scores ranging from 21 to 40 points. Distribution across categories reveals that the majority rated themselves as having high empathy: 65.0%, followed by 31.3% as moderate, and 3.7% as low (see Figure 1). The scale demonstrated acceptable internal consistency (Cronbach’s α = 0.76; Table 4), indicating a moderate level of reliability. Given that this value reflects only a moderate level of reliability, inferences regarding inter-item relations should be interpreted with caution.

**Figure 2:**
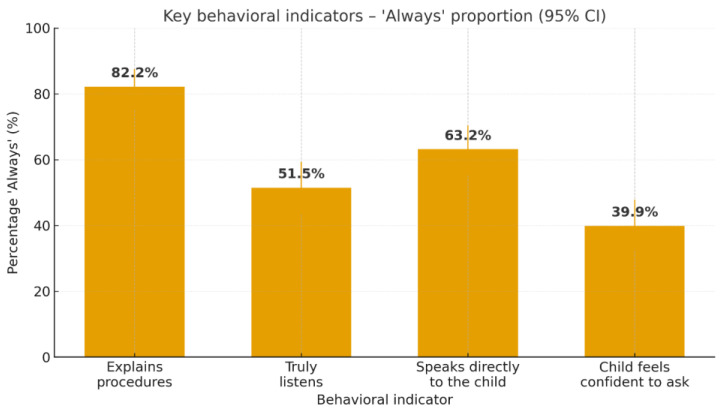
Proportion of ‘Always’ for key items.

At the behavioral level, the frequencies of the “always” response highlight the most visible areas of empathy in consultations: explaining procedures (82.2%), speaking directly to the child (63.2%), truly listening (51.5%), and the child feeling confident to ask questions (39.9%); notably, 30.7% of parents report “never” for the last indicator (Figure 2). Reliability details are presented in Table 5. Descriptive statistics for items (means and SD) are presented in Table 6. 

### 3.3. Differences Between Groups

**Figure 3:**
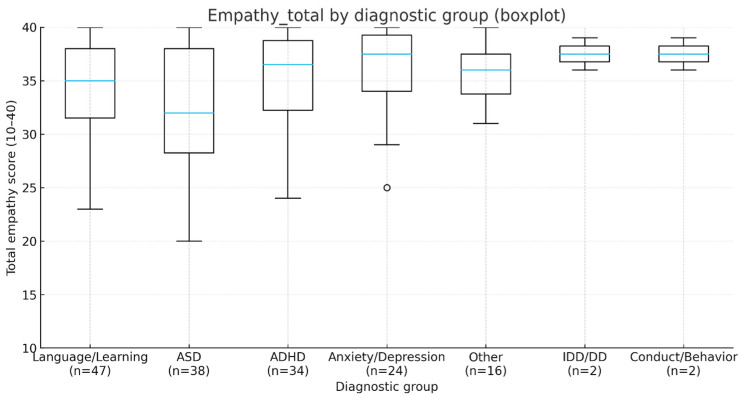
Boxplot of total empathy score by diagnostic group. The circle marks an outlier (defined as a value lying >1.5 × IQR beyond the box). The horizontal line inside each box indicates the median; whiskers extend to the most extreme non-outlier values. Diagnostic groups (*n*): Language/Learning (*n* = 47), ASD (*n* = 38), ADHD (*n* = 34), Anxiety/Depression (*n* = 24), Other (*n* = 16), IDD/DD (*n* = 2), Conduct/Behavior (*n* = 2). Total empathy score range: 10–40.

Analysis of variability in empathy scores across clinical and demographic characteristics showed a significant effect of diagnosis. In a one-way ANOVA comparing ASD, ADHD, anxiety/depression, and language/learning, there was an overall group difference, F(3, 139) = 4.06, *p* = 0.008, η^2^ = 0.08. Bonferroni-adjusted pairwise tests indicated lower scores in ASD versus anxiety/depression (mean difference −3.91; 95% CI −6.43 to −1.40; *p*_Bonf = 0.017), whereas all other contrasts were non-significant. Given the modest subgroup sizes (e.g., ASD, *n* = 38; anxiety/depression, *n* = 24) and the use of multiplicity correction, between-group differences should be interpreted as tentative. Means and standard deviations by diagnosis are summarized in Table 7, and distributions are displayed in Figure 3. Pairwise post hoc comparisons are presented in Table 8.

Pairwise comparisons using Welch t-tests with Bonferroni correction (6 comparisons) showed that parents of children with ASD reported significantly lower total empathy scores than parents of children with anxiety/depressive disorders (mean difference = −3.914; 95% CI −6.425 to −1.404; t (≈59.71) = −3.119; raw *p* = 0.003; *p*_Bonf = 0.017). All other pairwise contrasts were not statistically significant after Bonferroni correction. The ASD vs. anxiety/depressive contrast corresponds to a moderate–large effect size (Cohen’s d ≈ −0.75), indicating a clinically meaningful difference in perceived empathy between these groups.

No statistically significant differences in total empathy scores were observed between parents of different sexes (t = 1.71, *p* = 0.104). Likewise, residence (urban vs. rural) was not associated with empathy scores (t = −1.33, *p* = 0.186). Parental educational level did not predict differences in perceived physician empathy (one-way ANOVA, F(4, 158) = 0.69, *p* = 0.598).

### 3.4. Correlations and Models

A small but statistically significant positive correlation was observed between child age and parent-perceived physician empathy (Pearson’s r = 0.168; 95% CI [0.015, 0.314]; *p* = 0.032; N = 163). Additionally, the child’s state at the end of the consultation correlated moderately and significantly with perceived empathy (Spearman’s ρ = 0.427; 95% CI [0.292, 0.545]; *p* < 0.001; N = 163). These findings indicate that, on average, older children are perceived to receive slightly higher levels of physician empathy, and that a calmer/more positive state at discharge is associated with higher parent-rated empathy during the visit (See Table 9).

#### Multivariable Linear Regression

To examine the simultaneous influence of the predictors, a multiple ordinary least squares (OLS) regression was fitted, with the total perceived empathy score as the dependent variable and child diagnosis (ADHD as the reference) and child age as predictors. The model explained 10.0% of the variance in empathy scores (R^2^ = 0.100). The model’s modest explanatory power (R^2^ ≈ 0.10) suggests that unmeasured contextual factors likely account for additional variance. Compared with ADHD, autism spectrum disorder (ASD) was associated with a significantly lower empathy score (β = −2.199; SE = 0.874; 95% CI −3.926 to −0.472; *p* = 0.013). Child age had a small but positive effect (β = +0.159 points per year; SE = 0.075; 95% CI 0.011 to 0.306; *p* = 0.035). The remaining diagnostic categories were not statistically significant (*p* > 0.05). The analysis used classical standard errors and was performed on complete cases (See Table 10).

### 3.5. Principal Component Analysis (PCA)

The dataset met only borderline conditions for factor analysis (near-singular correlation matrix; complete-case subset KMO = 0.54; Bartlett χ^2^(10) = 17.6, *p* = 0.062). Internal consistency was acceptable (α = 0.73).

**Figure 4:**
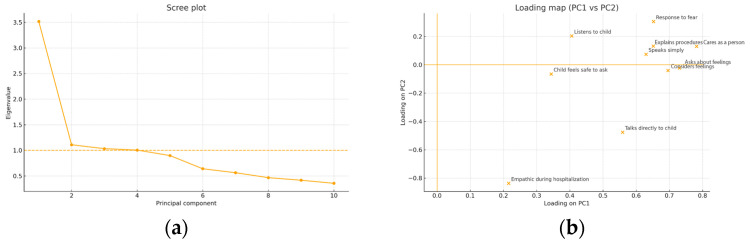
(**a**) Scree plot (10 items; MICE-imputed): pronounced elbow after PC1; dashed line marks eigenvalue = 1. (**b**) Loading map (PC1–PC2): items cluster along a “communication clarity/direct address” axis (PC1) vs. a “facilitation/participation” axis (PC2).

A PCA with varimax rotation suggested a three-factor solution (eigenvalues > 1, scree plot, interpretability) explaining 78.5% of variance (Table 11; Figure 4). Factors reflected: (i) direct, child-centered empathic communication; (ii) facilitation of child participation; and (iii) empathic listening. Three items (pain reporting, care for the child as a person, empathy during hospitalization) showed negligible communalities, limiting their factorial contribution (Table 12).

Given borderline sampling adequacy and unstable loadings for some items, these findings should be regarded as exploratory. The PCA offers tentative insights into possible subdimensions of parent-perceived empathy, but requires replication with refined items and larger, independent samples.

Because several items exhibited near-constant distributions, the full 10-item correlation matrix was nearly singular, precluding stable KMO/Bartlett estimation. Consequently, PCA results are interpreted with caution and were cross-checked against a complete-case subset with adequate variability (KMO = 0.538; Bartlett χ^2^(10) = 17.605, *p* = 0.062).

## 4. Discussion

Our findings should be interpreted within the specific context of outpatient child and adolescent psychiatry. Where caregiver mediation is pervasive and symptom profiles (e.g., avoidance, cognitive rigidity, heightened arousal) render empathic behaviors not merely desirable but integral to therapeutic engagement. Compared with general pediatrics, these consultations more often involve elevated anxiety, communication differences, and a need for predictable, low-stimulus interactions—features that shape both the enactment and the perception of empathy, especially when communication is filtered through caregivers. In this setting, higher parent-perceived empathy was associated with a calmer, more understood end-of-visit state for the child, a pattern consistent with prior work linking expressed clinician empathy to patient satisfaction and perceived care quality, including evidence from randomized trials and meta-analytic syntheses supporting empathy-enhancement interventions [26]. Brief pre-consultation compassion prompts have likewise been shown to reduce patient anxiety, offering a plausible mechanism for the observed association [26,27,28,29].

The link between empathy and a calmer departure state should be viewed as correlational, not causal. Reverse causality is plausible—children who leave the visit calmer may lead parents to perceive greater empathy from the clinician. While the directionality aligns with evidence that empathic communication attenuates anxiety, our cross-sectional design precludes inference about temporal or causal pathways.

Evidence from pediatric intensive care conferences indicates that when physicians deliver unburied empathetic statements and then pause, families are dramatically more likely to share emotional concerns (OR 18; 95% CI 10.1–32.4; *p* < 0.001), underscoring that not just empathy—but the timing and delivery of it—is critical for fostering parent-clinician connection [30].

Recent research among pediatricians revealed that high outpatient volume and communication constraints can negatively affect empathy expression; conversely, smoother interactions were observed in settings like rheumatology and general surgery, suggesting that clinical context and workload significantly modulate empathic behavior [31].

To ensure logical consistency, we revisit our objectives in the same order as introduced: (i) overall empathy levels, (ii) subgroup differences, (iii) correlational patterns, (iv) independent predictors, and (v) dimensionality of the empathy scale. First, we found high mean empathy scores, yet with evident gaps in active listening and in encouraging questions. Second, regarding subgroup differences, only the ASD vs. anxiety/depression contrast remained significant after correction, while all other differences should be considered tentative. Third, empathy correlated modestly with child age and moderately with the child’s end-of-visit calmness. Fourth, the regression explained approximately 10% of the variance, suggesting that relevant contextual variables were not captured. Finally, PCA indicated a general empathy factor and potential subdimensions, but with borderline adequacy and several items showing near-zero communalities.

The convergence between our findings and prior literature on observable communication behaviors is noteworthy. Studies in pediatric outpatient settings consistently highlight a set of strategies that positively shape families’ perception of communication quality: explaining the next steps, using simple language, actively soliciting questions, and addressing the child directly whenever possible. Data from pediatric intensive care consultations also demonstrate that “unburied” empathic statements—followed by a deliberate pause—facilitate therapeutic alliance and disclosure of family values and concerns. This procedural detail offers a plausible explanation for why active listening and encouraging questions emerged in our sample as weaker yet high-yield targets for improvement [30,32].

Our finding of lower empathy scores in children with ASD is consistent with contemporary literature emphasizing the need for specific adaptations in clinical interaction to reduce sensory load and increase predictability. Recommended strategies include flexible scheduling, stepwise explanations supported by visual aids, additional processing time, and the use of literal, direct language. Recent reviews underline those barriers related to the “double empathy problem” and atypical communicative demands may erode the perception of empathy in the absence of such adaptations, even when clinicians’ intentions are empathic. These observations provide context for the negative coefficient of the ASD diagnosis in our OLS model [33,34,35]. Our findings of diagnosis-related differences in parent-perceived empathy, with lower scores particularly in children with ASD, resonate with broader psychiatric literature emphasizing the role of symptom severity and clinical complexity as predictors of patient–clinician interaction quality. Recent work in adult schizophrenia has highlighted that both predictive factors and the spectrum of symptom severity strongly influence care experiences and management needs, underscoring the importance of adapting physician communication to diagnostic profiles [36].

While lower empathy ratings in ASD are consistent with previous literature, alternative explanations should be acknowledged. Parental expectations of additional accommodations, caregiver stress, and the child’s communicative profile (e.g., atypical reciprocity, sensory sensitivities) may all influence how empathic behaviors are perceived and rated. The “double empathy problem” framework further emphasizes that communication breakdowns can be bidirectional, arising not solely from physician shortcomings but also from mismatched styles and expectations between autistic children and clinicians.

The clinical implications are immediate:

- Standardizing empathic behaviors through structured checklists (e.g., Kalamazoo/KEECC, m-SPIKES) and micro-behavioral steps (explain–listen–summarize–pause) appears both rational and feasible. Educational interventions for residents and students have demonstrated measurable gains in communication and empathy skills, including in randomized trials [37,38,39].
- For neurodivergent populations, “autism-friendly” protocols advocate environmental and communicative adaptations co-designed with service users; their implementation increases trust and reduces distress [33,35].
- Incorporating parent–child feedback into quality assessment via PREMs aligns with recent pediatric service recommendations, where patient- and family-reported measures are increasingly used as indicators of care quality [33,35,37,38,39,40].

At the educational and systems level, there is growing support for integrating structured training in clinical empathy and family communication into medical curricula. Recent reviews and meta-analyses show that empathy can be taught and maintained through active methods such as role-play, video-feedback, empathy portfolios, and deliberate practice. From a quality-management perspective, routine use of PROMs and PREMs in pediatrics has been associated with improvements in communication, shared decision-making, identification of unmet needs, and monitoring of intervention outcomes [1,37,41,42].

The strengths of our study include completeness of scale data, good internal consistency, and convergence of results across correlational analyses, OLS regression, and exploratory principal component analysis. Limitations relate to the single-center design and reliance on the parental (proxy) perspective, both well-discussed in pediatric PREM literature, where parent–child discrepancies may occur and warrant documentation. Future research should include children’s direct reports and triangulation with validated empathy-perception instruments (e.g., JSPE/JSPE-HP, JSPPPE, Visual CARE) [13,17,18,19]. We also did not measure consultation length, a potentially relevant variable for perceived empathy [40,43,44,45]. It is particularly important to stress that parent-only proxy data may diverge from children’s self-reported experiences; previous PREM research consistently documents discrepancies between informants, underscoring the necessity of multi-informant approaches.

This study has several limitations. First, it was conducted in a single outpatient child psychiatry clinic in Romania, which restricts generalizability to other cultural or service contexts. Second, the findings are based exclusively on parental (proxy) reports, omitting the perspectives of children and clinicians; prior PREM research shown consistent discrepancies between informants, underscoring the need to triangulate multiple viewpoints. Third, we did not prospectively capture the denominator of approached/eligible families, precluding calculation of a participation rate and preventing a formal assessment of selection bias. Fourth, the range of psychiatric diagnoses represented was limited, with small subsamples in several groups, reducing statistical power. Fifth, the empathy scale demonstrated only moderate internal consistency (Cronbach’s α = 0.76) and borderline factorability, with some items contributing limited discriminative variance; accordingly, dimensional inferences remain exploratory. Finally, we did not measure potentially important contextual confounders (e.g., consultation length, clinician-level factors, prior therapeutic relationship), which may account for additional variance in perceived empathy. These issues caution against overinterpretation and highlight the need for replication in larger, multi-center samples using validated, multi-informant tools.

### Future Directions Include

8. pragmatic trials of brief educational interventions to standardize empathic behaviors, assessed through PREMs;
9. integration of consultation duration and process indicators (e.g., the “pause after an empathic statement”);
10. triangulation of parent–child–clinician perspectives using validated instruments; and
11. evaluation of impact on child anxiety and family satisfaction [26,30].

## 5. Conclusions

Parent-perceived physician empathy was generally high in this outpatient pediatric psychiatry sample, with variation across diagnostic groups. Perceptions appear shaped not only by individual skills but also by contextual features of child mental-health care. Exploratory PCA provided tentative evidence of potential subdimensions—child-centered clarity/direct address and listening/facilitation—that warrant replication with larger samples and refined items. Clinically, visible empathic behaviors (active listening, stepwise explanations, direct engagement, facilitation of questions) should be prioritized. For autism, adaptations should also reflect the double empathy problem, emphasizing mutual accommodation (predictability, concrete language, additional processing time, reduced sensory load). Correlational and multivariable results indicated a modest association with child age and a link to a calmer, more understood end-of-visit state; these associations are non-causal and should be interpreted cautiously. Embedding structured training (e.g., checklists and micro-steps such as explain–listen–check understanding–encourage questions) and PREM-based feedback into routine practice may strengthen the therapeutic alliance and improve the care experience for children and families.

While empathy is globally perceived as good, its added value is realized through concrete behaviors and targeted adaptations, particularly for children with ASD. Training programs and systematic feedback can transform empathy from a declarative principle into an observable standard of care, with direct impact on the child’s emotional comfort and the family’s long-term collaboration.

### Clinical Implications

The present findings underscore that empathy in pediatric psychiatry is not merely an attitudinal construct but a set of observable behaviors that can be standardized, trained, and monitored. Embedding structured empathic behaviors into daily clinical routines—such as explaining next steps clearly, directly addressing the child, checking understanding, and encouraging questions—has the potential to improve both child comfort and family trust. For children with autism spectrum disorder, the incorporation of neurodiversity-specific adaptations (predictable sequencing, concrete language, visual supports, and sensory-friendly environments) is particularly important to ensure equitable care experiences.

At the systems level, routine integration of PREMs (Patient Reported Experience Measures) offers a feasible and family-centered approach to monitoring empathic communication as a quality indicator. From an educational standpoint, brief, structured training modules and feedback loops for residents and early-career clinicians can help translate empathy from a declared principle into an observable standard of care, thereby strengthening therapeutic alliance and long-term collaboration with families.

## Figures and Tables

**Table 1:** Sample characteristics.

Variable	Value
Child’s age (years)	10.35 ± 4.04
Residence	Urban 57.1%; Rural 42.9%
Parent’s gender	Women 91.4%; Men 8.6%

Note: Percentages are rounded to one decimal place and sum to 100% within each category.

**Table 2:** Distribution of parental education level.

Education Level	% of Total
No schooling	6.7
Primary	9.8
Lower secondary	21.5
Upper secondary	45.4
Tertiary	16.6

**Table 3:** Diagnostic Groups (Buckets).

Diagnostic Group	% of Total
Language/Learning	29.4%
ASD (Autism Spectrum Disorder)	23.3%
ADHD	20.9%
Anxiety/Depression	14.7%
Others	8.0%
IDD/DD (Intellectual/Developmental Delay)	2.5%
Conduct/Behavior	1.2%

**Table 4:** Cronbach’s α and reliability details.

Indicator	Value
Number of items	10
Cronbach’s α	0.76
Mean inter-item correlation (MIC)	0.28

**Table 5:** Cronbach’s α and Item Reliability Details.

Item	Corrected Item-Total Correlation	α If Item Deleted
Explains procedures	0.39	0.75
Truly listens	0.48	0.73
Speaks in an easy-to-understand way	0.46	0.74
Considers how the child feels	0.56	0.73
A child can say if something hurts (without fear)	0.45	0.74
Asks how the child feels (not just about the illness)	0.46	0.74
Shows care for the child as a person	0.50	0.74
The child feels brave enough to ask questions	0.46	0.74
Speaks directly to the child	0.41	0.74
How the child feels at departure	0.39	0.74

Note. MIC = mean inter-item correlation; r_item–total = corrected item-total correlation; α = Cronbach’s alpha.

**Table 6:** Means and standard deviations for empathy items.

Item	N Valid	Mean (1–4)	SD
Explains procedures	163	3.79	0.51
Really listens	163	3.25	0.88
Speaks in an easy-to-understand way	163	3.64	0.68
Takes into account how the child feels	163	3.74	0.55
Can tell if it hurts (without fear)	163	3.07	1.15
Asks how the child feels (not just about the illness)	163	3.75	0.53
Shows care for the child as a person	163	3.72	0.55
The child feels brave enough to ask questions	163	2.69	1.28
Speaks directly to the child	163	3.35	0.97
How the child feels when leaving	163	3.52	0.84

**Table 7:** Empathy by Diagnostic Group.

Diagnosis	N	Mean	SD
ASD	38	32.21	5.91
ADHD	34	35.15	4.34
Anxiety/Depressive	24	36.12	3.97
Language/Learning	47	34.38	4.19

ANOVA across the four diagnostic groups: F(3, 139) = 4.06, *p* = 0.008, η^2^ = 0.08. Note. Group order: ASD, ADHD, Anxiety/Depressive, Language/Learning. Mean and SD values refer to the total empathy score (range 10–40).

**Table 8:** Pairwise post hoc comparisons (Bonferroni)—total empathy score (10–40).

Group 1	Group 2	n1	n2	Mean1	Mean2	Diff (G1–G2)	t	df	*p* (Raw)	*p* (Bonferroni)	95% CI Lower	95% CI Upper	Sig (Bonf.)
ASD	ADHD	38	34	32.211	35.147	−2.937	−2.420	67.566	0.018	0.109	−5.358	−0.515	ns
ASD	Anxiety/Depressive	38	24	32.211	36.125	−3.914	−3.119	59.710	0.003	0.017	−6.425	−1.404	*
ASD	Language/Learning	38	47	32.211	34.383	−2.172	−1.911	64.678	0.060	0.362	−4.443	0.098	ns
ADHD	Anxiety/Depressive	34	24	35.147	36.125	−0.978	−0.888	52.265	0.378	1.000	−3.186	1.231	ns
ADHD	Language/Learning	34	47	35.147	34.383	0.764	0.793	69.771	0.431	1.000	−1.158	2.686	ns
Anxiety/Depressive	Language/Learning	24	47	36.125	34.383	1.742	1.715	48.758	0.093	0.556	−0.299	3.783	ns

Note. Welch tests for pairwise comparisons; Bonferroni adjustment for 6 comparisons. Significance code: * *p* < 0.05 (after Bonferroni); ns = not significant. *p*-values Bonferroni-adjusted; report mean differences with 95% CI and effect sizes (Cohen’s d/partial η^2^).

**Table 9:** Correlations with empathy.

Variable Pair	N	Coefficient	Type (r/ρ)	95% CI	*p* (Two-Tailed)
Empathy score vs. Child age (years)	163	0.168	r (Pearson)	[0.015, 0.314]	0.032
Empathy score vs. Child state at end of consultation	163	0.427	ρ (Spearman)	[0.292, 0.545]	<0.001

**Table 10:** OLS coefficients (β, SE, 95% CI, *p*).

Predictor	β	SE	95% CI	*p* (Two-Tailed)
ASD (vs ADHD, reference)	−2.199	0.874	[−3.926, −0.472]	0.013
Anxiety/Depressive (vs. ADHD)	+0.702	0.948	[−1.169, 2.574]	0.460
Language/Learning (vs. ADHD)	−0.300	0.818	[−1.915, 1.315]	0.714
Child age (years)	+0.159 per year	0.075	[0.011, 0.306]	0.035

Note. Dependent variable = total empathy score (range 10–40); OLS with conventional (classical) standard errors; R^2^ = 0.100; two-tailed tests; complete-case analysis.

**Table 11:** Eigenvalues and explained variance (10 items; MICE-imputed; N = 163).

Component	Eigenvalue	% Variance Explained	Cumulative %
PC1	2.95	41.9	41.9
PC2	1.56	22.2	64.1
PC3	1.01	14.4	78.5
PC4–PC10	<1.00	<10.9 each	100

Notes. Extraction of the correlation matrix. Retention by eigenvalue > 1 and scree. See Figure 4a.

**Table 12:** Varimax-rotated loadings and communalities (10 items; MICE-imputed; salient loadings by absolute magnitude).

Item (Short Label)	PC1	PC2	PC3
Uses child-friendly speech	**−0.89**	0.06	−0.18
Considers what the child feels	**−0.77**	0.10	−0.34
Asks how the child feels	**−0.81**	0.18	0.13
Talks directly to the child	**−0.60**	0.06	−0.16
Child dares to ask	−0.06	**0.92**	−0.36
Explains procedures (clarity)	0.13	**−0.97**	−0.19
Truly listens	−0.17	0.07	**−0.95**
Can report pain without fear	−0.00	−0.00	−0.00
Shows care for the child as a person	−0.00	−0.00	0.00
Empathic during hospitalization	0.00	0.00	0.00

Notes. Three components were retained and varimax-rotated. Loadings represent the strength of association between each item and the extracted components; values ≥ 0.40 (shown in **bold**) are typically considered meaningful. Signs are arbitrary under factor reflection, so interpretation is based on absolute magnitude. Given the borderline sampling adequacy and near-singular item correlations, these findings are exploratory and should be interpreted with caution.

## Data Availability

Due to GDPR requirements and the terms of the ethics approval, the individual-level dataset collected for this study cannot be shared publicly or upon request. Participant consent did not include data sharing beyond the research team, and institutional policy prohibits external redistribution of these data.

## Abbreviations

The following abbreviations are used in this manuscript:

ASD	Autism Spectrum Disorder
ADHD	Attention-Deficit/Hyperactivity Disorder
ANOVA	Analysis of Variance
CAMHS	Child and Adolescent Mental Health Services
CARE	Consultation and Relational Empathy (Measure)
OLS	Ordinary Least Squares
CI	Confidence Interval
HC3	Heteroskedasticity-Consistent Standard Errors, Type 3
PCA	Principal Component Analysis
IDD/DD	Intellectual and Developmental Disabilities/Developmental Delay
PREMs	Patient-Reported Experience Measures
PROMs	Patient-Reported Outcome Measures
JSPE	Jefferson Scale of Physician Empathy
SD	Standard Deviation
KMO	Kaiser–Meyer–Olkin
MIC	Mean Inter-Item Correlation
IRB	Institutional Review Board
GDPR	General Data Protection Regulation
